# Fish Protein Ingestion Induces Neural, but Not Muscular Adaptations, Following Resistance Training in Young Adults

**DOI:** 10.3389/fnut.2021.645747

**Published:** 2021-03-09

**Authors:** Kohei Watanabe, Aleš Holobar, Kenji Uchida, Yukiko Mita

**Affiliations:** ^1^Laboratory of Neuromuscular Biomechanics, Faculty of Liberal Arts and Sciences and School of International Liberal Studies, Chukyo University, Nagoya, Japan; ^2^Faculty of Electrical Engineering and Computer Science, University of Maribor, Maribor, Slovenia; ^3^Nihon Suisan Kaisha, Ltd., Tokyo, Japan; ^4^Department of Human Nutrition, School of Life Studies, Sugiyama Jogakuen University, Nagoya, Japan

**Keywords:** Alaska pollack protein, nutritional supplementation, multichannel surface electromyography, motor unit identification, neural adaptation

## Abstract

**Purpose:** Nutritional supplementation in conjunction with exercise is of interest for the prevention or improvement of declines in motor performances in older adults. An understanding of the effects on both young and older adults contributes to its effective application. We investigated the effect of fish protein ingestion with resistance training on neural and muscular adaptations in young adults using interventions and assessments that have already been tested in older adults.

**Methods:** Eighteen young adults underwent 8 weeks of isometric knee extension training. During the intervention, nine participants ingested 5 g of fish protein (*n* = 9, Alaska pollack protein, APP), and the other nine participants ingested casein as a control (*n* = 9, CAS) in addition to daily meals. Before, during, and after the intervention, the isometric knee extension force, lower extremity muscle mass, and motor unit firing pattern of knee extensor muscles were measured.

**Results:** Maximum voluntary contraction (MVC) was significantly increased in both APP and CAS groups from 0 weeks to 4, 6, and 8 weeks of intervention (*p* < 0.001), but there were no significant differences between the groups (*p* = 0.546–0.931). Muscle mass was not significantly changed during the intervention in either group (*p* = 0.250–0.698). Significant changes in motor unit firing rates (*p* = 0.02 and 0.029 for motor units recruited at 20–40% of MVC and at 40–60%) were observed following the intervention in the APP but not CAS (*p* = 0.120–0.751) group.

**Conclusions:** These results suggest that dietary fish protein ingestion changes motor unit adaptations following resistance training in young adults.

## Introduction

Protein supplementation enhances increases in muscle volume and strength following resistance training when the training regimen and nutritional program are adequate (1). Since protein supplementation improves muscle protein synthesis following resistance training (2), this enhanced gain in muscle strength can be explained by the enhanced muscle hypertrophy, which is categorized as muscular adaptation. Generally, neural adaptation also explains gains in muscle strength following resistance exercise, such as changes in motor unit firing properties and recruitment patterns (3–5). Moreover, besides providing direct exercise-induced neural adaptations, changes of peripheral muscle contractile properties following resistance exercise (6) may also induce neural adaptations as alternative pathways, since muscle contractile properties are key determinants of motor unit firing rate (7, 8). Therefore, protein supplementation may change not only muscular factors but also neural factors when performed in conjunction with resistance training. In fact, we previously reported a trade-off relationship between neural and muscular factors during intervention involving resistance training with protein supplementation. During 8 weeks of resistance training intervention, older adults who ingested fish protein showed gains in muscle strength with muscle hypertrophy but without marked motor unit adaptation, while older adults who ingested casein protein showed similar gains in muscle strength with marked motor unit adaptation but without muscle hypertrophy (9). Since previous studies already reported that fish protein intake induced greater muscle hypertrophy when compared with casein intake in rat muscle, enhancements of muscle hypertrophy following resistance training by the ingestion of fish protein in older adults may be likely to be reasonable (10–12).

Contributions of neural and muscular factors to muscle strength gain during resistance training differ between young and older adults. Older adults show relatively stronger and weaker contributions of neural and muscular factors, respectively, to gains in muscle strength during resistance training intervention compared with young adults (13–15). The maximal motor unit firing rate increased by 15% in young adults and 49% in older adults following a 6-week resistance exercise training program (14). While muscle protein synthesis after resistance training is impaired in older adults (16), their ingestion of essential amino acids after resistance training also enhances muscle protein synthesis (17). From these findings, we hypothesized that neural and muscular adaptations would differ even when applying the same exercise and nutritional intervention between young and older adults.

The purpose of this study was to investigate the effect of fish protein ingestion on neural and muscular adaptation during resistance training intervention in young adults in order to clarify the age-specific neural and muscular adaptations in combinations of nutritional supplementation and resistance training. We hypothesized that the effect of fish protein ingestion on neural and muscular adaptations following resistance training would be different in young adults when compared with the results for older adults obtained in a previous study (9), because the contribution and/or sensitivity of neural and muscular adaptations to resistance training-induced muscle strength gain (3–5) and role of muscle protein synthesis with nutritional supplementation are markedly influenced by aging (16, 17).

## Materials and Methods

### Participants

Eighteen healthy young males and females participated in this study (Table 1). The participants gave written informed consent after receiving a detailed explanation of the purposes, potential benefits, and risks associated with participation. This study was approved by the Research Ethics Committee of Chukyo University (2015-002, 2016-057) and conducted in accordance with the Declaration of Helsinki.

**Table 1:** Characteristics of the participants.

**Group**	**APP**	**CAS**	***p*-values for comparison between the groups**
*n*	9	9	
*n* of males	6	7	
Age (years)	21.6 ± 0.5	21.7 ± 0.5	0.730
Height (cm)	165.8 ± 7.8	169.5 ± 9.4	0.387
Body mass (kg)	53.9 ± 9.2	58.5 ± 6.1	0.436
Muscle mass (kg)	25.7 ± 5.0	28.5 ±5.1	0.489
Fat mass (kg)	7.6 ± 2.6	7.8 ± 3.1	0.666
Lower extremity muscle mass (kg)	7.5 ± 1.5	8.4 ± 1.6	0.340
MVC (N)	516.9 ± 171.7	559.8 ± 128.0	0.666

*APP, Alaska pollack protein ingestion; CAS, casein ingestion*.

### Resistance Training

During 8-week intervention, all participants trained their left and right knee extensor muscles using isometric unilateral knee extension resistance training twice a week. The contraction intensity was >80% of their maximal voluntary contraction (MVC) torque, contraction time was 4 s of the sustained phase at or over the target torque with a 1-s increasing phase to the target torque, training volume was 3 sets of 10 contractions for each leg, and rest intervals between contractions and sets were 10 and 60~120 s, respectively. During training, the performed and target torques were shown on a monitor to provide visual feedback and an experimenter confirmed the performed torque and encouraged the participants to complete the training program. After the measurements of MVC every 2 weeks, the target torque was revised when MVC increased. The training was performed using a custom-made dynamometer (Takei Scientific Instruments Co., Ltd., Niigata, Japan) with a force transducer (LU-100KSE; Kyowa Electronic Instruments, Tokyo, Japan). The same training regimen was administered to older adults in our previous study (9).

### Nutritional Supplementation

The participants were divided into two groups of nine participants who ingested 5 g of fish protein (Alaska pollack protein, APP) or 5 g of casein as a control (CAS) in addition to normal daily diets. Amino acid scores for APP and CAS were both 100 and the detailed amino acid components of each protein are described previously (11). The participants took them in a cup of soup every day with either breakfast, lunch, or dinner. A cup of soup for APP/CAS was comprised of 5.2 of protein including 5 g of APP/CAS, 0.5/0.6 g of fat, and 10.7/10.2 g of carbohydrate. Total energy content of soup in APP and CAS was 68 and 67 kcal, respectively. Amounts of protein supplementations as a function of the body mass of participants for APP and CAS were 0.095 ± 0.016 and 0.086 ± 0.010 g/kg/d, respectively. These values of APP supplementation were calculated from the results of previous studies using rats (10, 11). On the other hand, the amount of CAS supplementation used in this study is relatively low in comparison with amounts used in previous studies (10–63 g/d or 0.3 g/kg/d) (18, 19). This amount of CAS could be used as a control condition since it may not be sufficient to act intrinsically via protein supplementation on muscle strength and mass. In fact, our previous study showed that ingestions of the same amounts of APP and CAS induced significant increases and no significant changes, respectively, in the muscle volume during 8 weeks of resistance training intervention in older adults (9). This suggests that these amounts of APP and CAS can lead to different muscular adaptations in each group after initiating resistance training. Nutritional supplementations of APP and CAS were applied as a randomized, double-blind, placebo-controlled treatment. To blind the study, we packed the soups for both conditions in powdered form in identical bags and the flavorings were controlled for each condition by a professional food company. A dietary survey was performed by a nutritionist with a license from the Ministry of Health, Labor and Welfare in Japan, using a brief-type self-administered diet history questionnaire (BDHQ) (20) to quantify total energy, carbohydrate, protein, and fat in daily diets and normalized values by individual body mass in order to estimate the participants' nutritional conditions during the intervention. This questionnaire was applied at around 6~8 weeks of intervention, and so the results should reflect nutritional conditions during the intervention.

### Measurements

During the 8-week intervention, the muscle strength, motor unit firing pattern, and anthropometry tests were conducted every 2 weeks: the day before the intervention commenced (0 wks), and 2 (2 wks), 4 (4 wks), 6 (6 wks), and 8 weeks (8 wks) after beginning the intervention. For familiarization with the motor tasks used in the measurements, the participants came to the laboratory before the first day of intervention.

Muscle strength of knee extensor muscles was measured based on MVC during isometric knee extension for the right leg using a custom-made dynamometer which was used in resistance training in the present study (see *Resistance training* for details). During MVC, participants' hip and knee joint angles were fixed at 90° (180° corresponds to full extension). The MVC trial consisted of 2–3 s of a gradual increase in knee extension torque to maximum effort and 2–3 s of a maintained plateau phase at maximum effort with a verbal count given at 1-s intervals. Two MVC trials were performed with a ≥ 2- min rest interval. The higher MVC from the two MVC trials was chosen and used in further analysis and as a reference to set a target force in resistance exercise. These MVC measurement procedures were determined according to the methods and equipment used in our previous studies (9, 21, 22).

After MVC trials, the participants performed unilateral submaximal isometric right knee extension ramp contraction from 0 to 30% of MVC (Ramp 30) and 0 to 70% of MVC (Ramp 70). These slow ramp contractions were applied in order to identify the motor unit recruitment threshold and calculate motor unit firing rates at various force levels. Ramp 30 consisted of a 15-s increasing phase from the baseline to 30% of the MVC force level with a 2% MVC/s rate of force increase and 15-s sustained phase at a 30% MVC force level. Ramp 70 consisted of an 18-s increasing phase from the baseline to 70% MVC force level with a 5% MVC/s rate of force increase. From two trials, that showing a smaller difference between target and performed torques was chosen for further analysis for Ramp 30 and Ramp 70, respectively.

Muscle mass of the whole body and lower limb was estimated from body impedance measurements (InBody270, InBodyJapan Inc., Tokyo, Japan) and body and fat masses were also measured. This method was used in our previous study and could detect increases in the muscle volume following resistance training with APP ingestion in older adults (9). Our previous study performed this body impedance method on different days over 3 weeks in fifty older male and female in order to test its reproducibility (22). The intraclass correlation coefficient (ICC) was 0.992 for muscle mass measured by this body impedance method. We thus believe that this body impedance method could provide detectable and reliable changes in muscle mass following the intervention.

### Motor Unit Decomposition

During the submaximal ramp contractions, multi-channel surface EMG signals were recorded from the vastus lateralis (VL) muscle of the right leg using a semi-disposable adhesive grid of 64 electrodes (13 rows and 5 columns with one missing electrode at the corner) with a 1-mm diameter and 8-mm inter-electrode distance (ELSCH064NM2, OT Bioelettronica, Torino, Italy). Prior to attaching the electrodes, the skin was shaved and cleaned by alcohol. Conductive gels were inserted into the cavities of the electrode grid to assure proper electrode-skin contact. Monopolar surface EMG signals were recorded by a multi-channel surface EMG amplifier (quattrocento, OT Bioelettronica, Torino, Italy) and amplified by a factor of 150, sampled at 2,048 Hz, and converted to digital form by a 16-bit analog-to-digital converter together with the force transducer signal. The center of the electrode grid was located at the mid-point of the longitudinal axis of the VL muscle, i.e., the line between the head of the greater trochanter and inferior lateral edge of the patella, and the columns were aligned along the VL longitudinal axis. A reference electrode was placed at the right iliac crest. These electrode locations were the same as in our previous studies (9, 21, 22).

From the recorded multi-channel surface EMG signals, individual motor unit firing patterns were decomposed by the Convolution Kernel Compensation (CKC) technique (23–26). We used the decomposition procedure that was previously extensively validated for signals from various skeletal muscles (21, 26, 27) and used the pulse-to-noise ratio (PNR), introduced by Holobar et al. (28), as an indicator of the motor unit identification accuracy (28). Only motor units with PNR > 30 dB, corresponding to an accuracy of motor unit firing identification > 90%, were used for further analysis; all other motor units were discarded (28). After decomposition, we performed visual inspection to identify discharge times for individual motor units based on PNR, and the discharge times were used for calculation of instantaneous motor unit firing rates for individual motor units. We excluded the discharges with inter-discharge intervals <33.3 or >250 ms, since firing rates calculated from this range of inter-discharge intervals are unusually high (>30 Hz) or low (<4 Hz) for the VL muscle (21, 26).

Median firing rates of individual motor units were calculated from instantaneous firing rates during each 5% of MVC for Ramp30 and 10% of MVC for Ramp70. For example, the median firing rate at 15% of MVC for Ramp30 was calculated from the instantaneous firing rates in the interval from 12.5 to 17.5% of MVC, and the median firing rate at 50% of MVC for Ramp70 was calculated from instantaneous firing rates in the 45–55% MVC interval. Median firing rates with >30% coefficient of variation were excluded for further analysis (29). We divided the detected motor units into three groups by the recruitment force: motor units recruited at <10% (MU10), 10–20% (MU20), and 20–30% of MVC (MU30) for Ramp30, and 0-20% (MU20), 20–40% (MU40), and 40–60% of MVC (MU60) for Ramp70. Averaged values were calculated for each motor unit group with varying recruitment thresholds in different intervention periods. It is well-known that firing patterns are inconsistent among motor unit groups with different recruitment thresholds (30, 31). Our previous studies demonstrated recruitment threshold-dependent changes in motor unit firing patterns following resistance training (9, 22). Also, since the relationship between motor unit firing rate and exerted force is not linear (21, 32), firing rate should be analyzed at various force levels in order to assume the detailed neural adaptations. Therefore, the present study performed analyses of individual motor unit groups with different recruitment thresholds at various force levels. These procedures for calculating motor unit firing rates were employed in our previous studies (9, 21, 22).

### Statistics

The results are reported as the mean ± SD. To determine whether data showed a normal distribution, we performed the Shapiro-Wilk test before statistical analysis. We decided to use non-parametric statistical tests, since our results included non-normally distributed data and the sample size was not large enough to use parametric analysis.

For comparisons of age, height, body mass, muscle mass, fat mass, and lower limb muscle mass estimated by InBody, and MVC between the groups at 0 wks, the Mann-Whitney test was used. The body mass, muscle mass, fat mass, and lower limb muscle mass estimated by InBody, and MVC at 2, 4, 6, and 8 wks were normalized by the values at 0 W. The normalized values were analyzed by the Friedmann test to assess the effect of intervention, and the Bonferroni-Dunn test was used to compare the values at 2, 4, 6, and 8 wks with the value at 0 wks when a significant effect of intervention was detected (33). The Mann-Whitney test was performed to compare the nutritional parameters calculated from BDHQ between the groups. We also compared the change in absolute values (Δ) from 0 to 2, 4, 6, and 8 wks in body mass, muscle mass, fat mass, and lower limb muscle mass, and MVC by Mann-Whitney test.

Motor unit firing rates for each force level and motor unit group in different time periods were tested by the Kruskal-Wallis test to analyze the effect of intervention. Dunn's test was used to compare the values at 2, 4, 6, and 8 wks with the value at 0 wks when a significant effect of intervention was detected by the Kruskal-Wallis test (34). Motor unit firing rates at each force level were also compared among the motor unit groups with different recruitment forces by the Kruskal-Wallis test. When this test showed a significant effect of the motor unit group, firing rates at a specified force level were compared among the motor unit groups with different recruitment forces by Dunn's test (34).

Statistical analysis was performed using SPSS (version 21.0, SPSS, Tokyo, Japan). Since we used multiple comparisons in *post-hoc* tests, the level of significance was partly modified by Bonferroni correction to minimize the familywise error rate and maintain statistical power (35). The modified level of significance was 0.05/the numbers of comparisons for each analysis. Therefore, for example, the level of significance in the *post-hoc* test to compare the variables among the periods was set at 0.0125 (0.05/4) when the variables at 2, 4, 6, and 8 wks were compared with 0 wks (4 pairs).

## Results

### Muscle Strength and Anthropometric Tests

**Figure 1:**
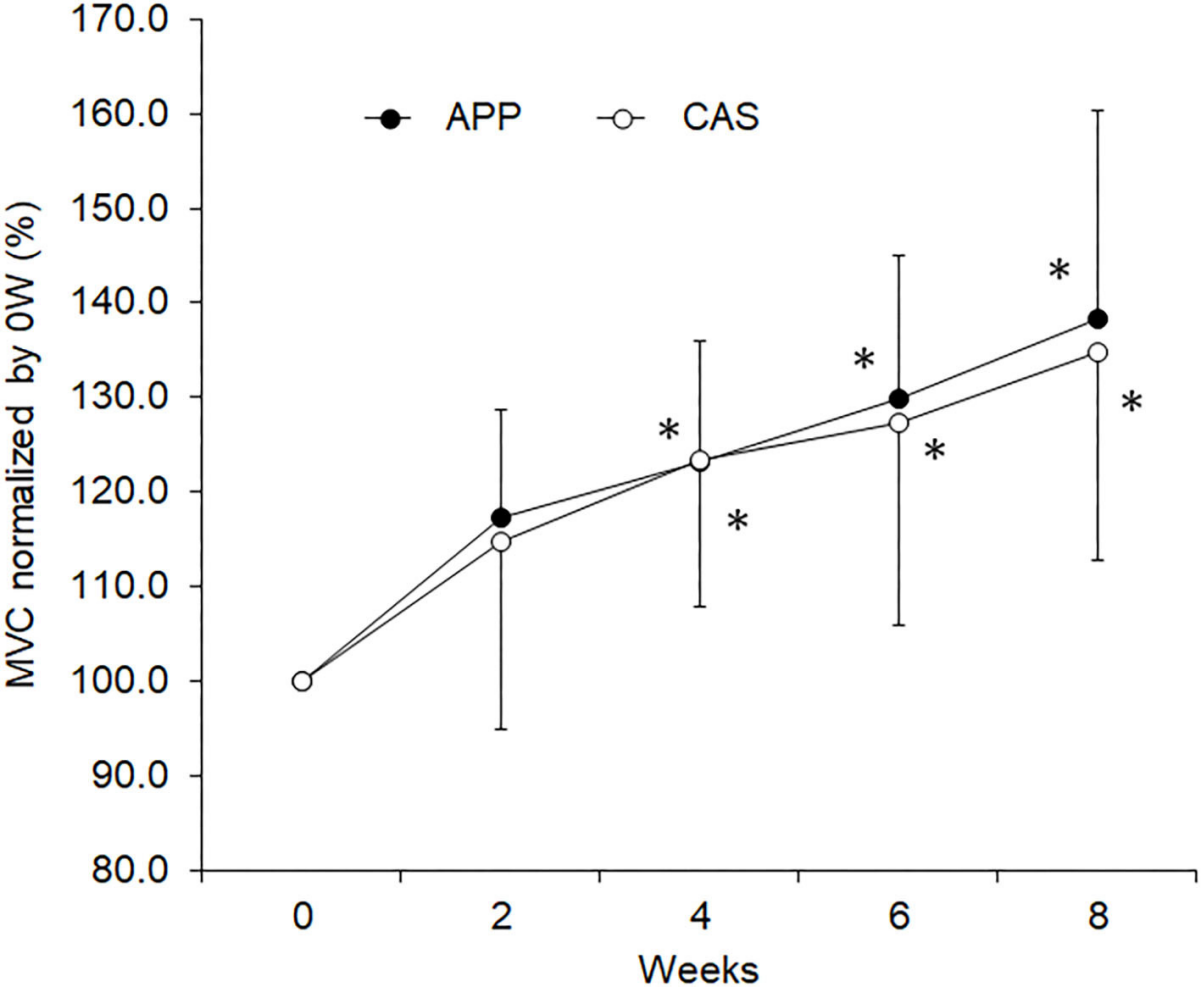
Maximal voluntary contraction (MVC) during isometric knee extension for the groups with ingestion of Alaska pollack protein (APP) and casein (CAS). The symbol * indicates a significant change from baseline (0 week)(*p* < 0.05).

No significant differences were observed in body mass, muscle mass, fat mass, or lower limb muscle mass estimated by InBody and MVC at 0 wks (Table 1) nor in their normalized values at 2, 4, 6, or 8 wks between APP and CAS groups (Table 2; *p* > 0.05). We detected a significant effect of intervention on MVC in both APP and CAS groups (*p* < 0.05), and MVCs at 4, 6, and 8 wks were significantly greater than those at 0 wks in both APP and CAS groups (*p* < 0.0125; Figure 1, Table 2). There were no significant effects of intervention on body mass, muscle mass, fat mass, or lower limb muscle mass estimated by InBody (Table 2; *p* > 0.05). No significant differences were found in changes in absolute values (Δ) from 0 to 2, 4, 6, and 8 wks in body mass, muscle mass, fat mass, and lower limb muscle mass, and MVC (*p* > 0.05; Table 2).

**Table 2:** Absolute and normalized values of anthropometric parameters and muscle strength.

		**APP**				**CAS**			
	**Weeks**	**Absolute**	**Δ**	**Normalized**	***p***	**Absolute**	**Δ**	**Normalized**	***p***
Body mass	0	53.9 ± 9.2		100.0 ± 0.0		58.5 ± 6.1		100.0 ± 0.0	
(kg)	4	54.0 ± 10.0	0.1	100.0 ± 2.3		58.8 ± 6.3	0.3	100.5 ± 0.7	
	8	53.6 ± 9.7	−0.3	99.3 ± 2.1		58.3 ± 6.2	−0.2	99.7 ± 1.4	
Muscle mass	0	25.7 ± 5.0		100.0 ± 0.0		28.5 ± 5.1		100.0 ± 0.0	
(kg)	4	25.8 ± 5.2	0.0	100.0 ± 1.9		28.8 ± 5.2	0.3	101.1 ± 1.3	
	8	25.7 ± 5.3	−0.1	99.6 ± 2.1		28.6 ± 5.0	0.2	100.7 ± 1.2	
Lower limb muscle mass	0	7.5 ± 1.5		100.0 ± 0.0		8.4 ± 1.6		100.0 ± 0.0	
	4	7.5 ± 1.6	0.0	100.6 ± 2.5		8.4 ± 1.6	0.0	100.3 ± 2.4	
(kg)	8	7.5 ± 1.5	0.0	100.0 ± 1.4		8.4 ± 1.6	0.0	99.7 ± 1.8	
Fat mass	0	7.6 ± 2.6		100.0 ± 0.0		7.8 ± 3.1		100.0 ± 0.0	
(kg)	4	7.6 ± 3.1	0.0	97.1 ± 19.2		7.6 ± 3.3	−0.2	96.7 ± 8.1	
	8	7.4 ± 2.6	−0.2	96.7 ± 14.2		7.4 ± 3.3	−0.4	94.3 ± 12.7	
MVC	0	516.9 ± 171.7		100.0 ± 0.0		559.8 ± 128.0		100.0 ± 0.0	
(N)	2	604.9 ± 203.9	88.0	117.3 ± 11.3		646.7 ± 184.9	87.0	114.7 ± 19.8	
	4	632.7 ± 209.2	115.8	123.2 ± 12.7	0.020	690.8 ± 176.2	131.0	123.3 ± 15.5	0.024
	6	667.2 ± 218.8	150.2	130.0 ± 15.1	0.004	717.0 ± 198.0	157.3	127.3 ± 21.4	0.016
	8	696.7 ± 189.2	179.7	138.3 ± 22.0	0.004	756.2 ± 203.0	196.4	134.7 ±21.9	0.004

*APP, Alaska pollack protein ingestion; CAS, casein ingestion*.

*p-values indicate post-hoc comparisons to baseline (0 weeks) in normalized value*.

### Nutritional Parameters

There were no significant differences in total energy (1,938.0 ± 602.1 kcal for APP and 1,988.5 ± 704.7 kcal for CAS), carbohydrate (279.5 ± 100.4 g for APP and 279.4 ± 92.2 g for CAS), protein (68.7 ± 26.4 g for APP and 71.3 ± 37.6 g for CAS), or fat (53.8 ± 20.6 g for APP and 58.0 ± 26.1 g for CAS) in daily diets and the values normalized by individual body mass in total energy (36.9 ± 12.9 kcal/kg for APP and 34.3 ± 12.0 kcal/kg for CAS), carbohydrate (5.2 ± 1.7 g/kg for APP and 4.8 ± 1.6 g/kg for CAS), protein (1.3 ± 0.6 g/kg for APP and 1.2 ± 0.6 g/kg for CAS), and fat (1.1 ± 0.5 g/kg for APP and 1.0 ± 0.4 g/kg for CAS) in daily diets between APP and CAS groups (*p* > 0.05 for all).

### Motor Unit Firing Patterns

In total, 1,048 motor units were identified in this study: 396 in APP and 344 in CAS groups for Ramp 30 and 161 in APP and 147 in CAS groups for Ramp 70.

**Figure 2:**
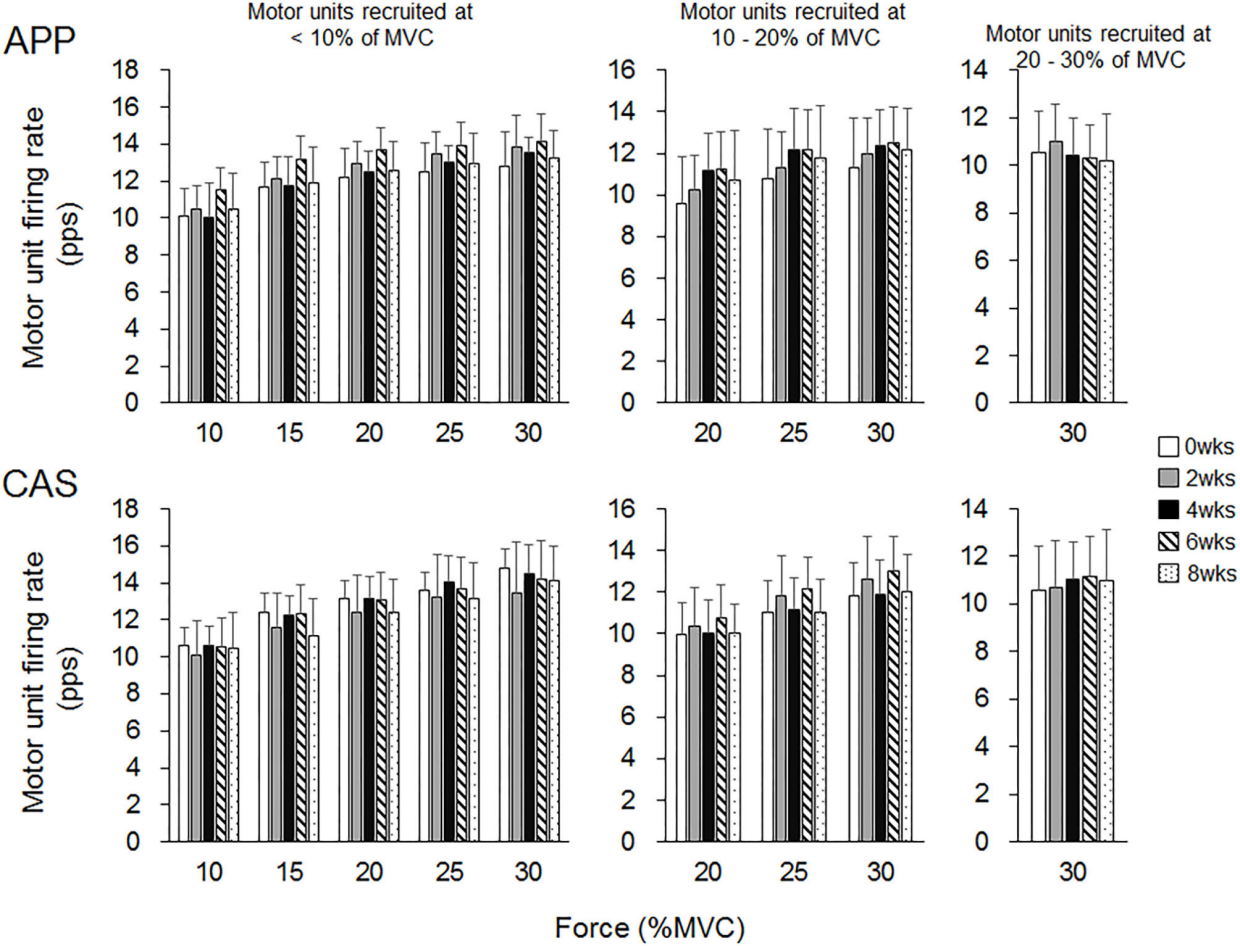
Motor unit firing rates during Ramp 30 in groups with ingestions of Alaska pollack protein (APP) and casein (CAS).

**Figure 3:**
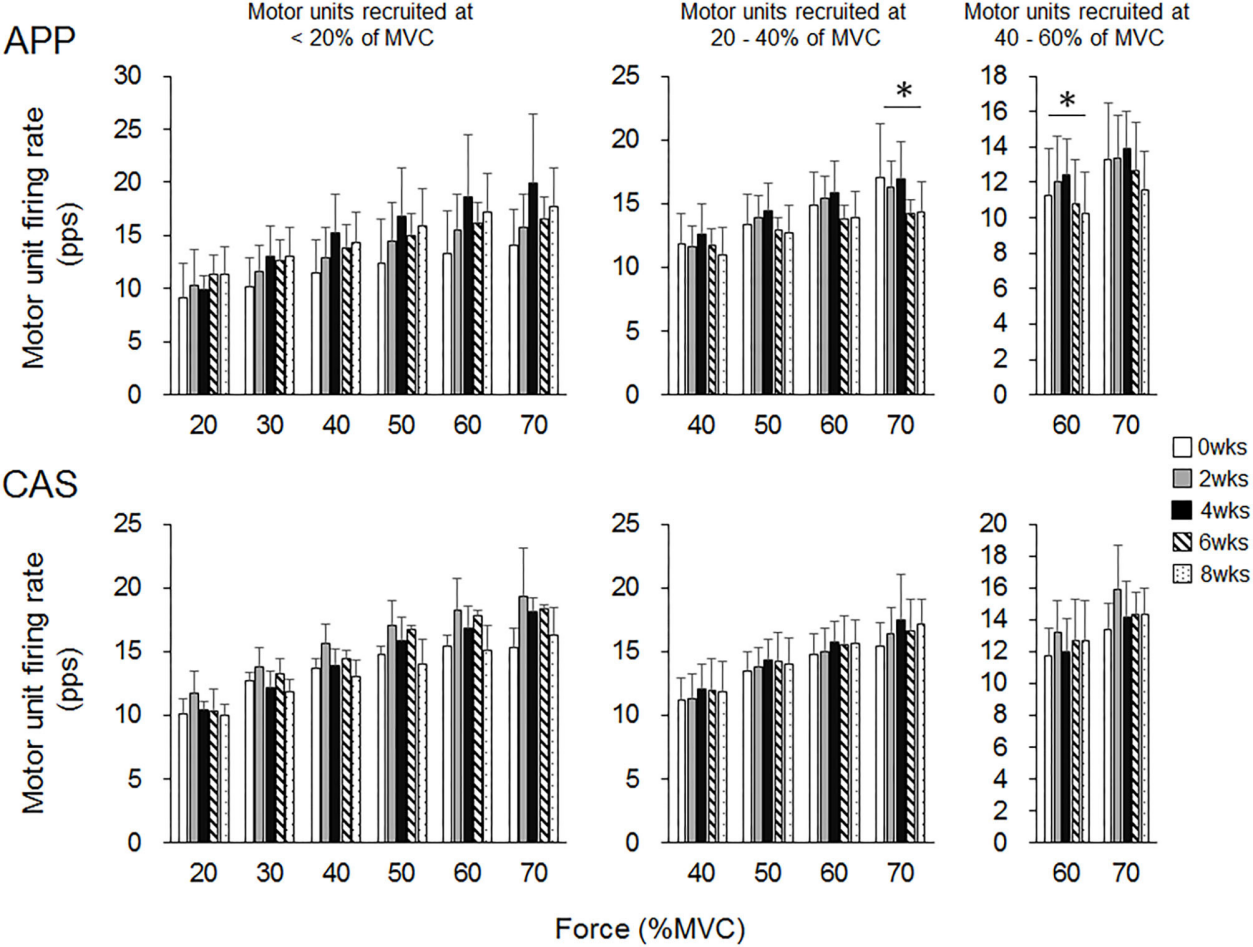
Motor unit firing rates during Ramp 70 in groups with ingestions of Alaska pollack protein (APP) and casein (CAS). The symbol * indicates a significant effect of intervention (*p* < 0.05) among the periods.

There were no significant changes in motor unit firing rates following the intervention for Ramp 30 in either the APP or CAS group (*p* > 0.05; Figure 2). No significant changes were noted in motor unit firing rates following the intervention for Ramp 70 in the CAS group (*p* > 0.05; Figure 3). In the APP group, significant changes in firing rates for MU40 and MU60 were noted at 70 and 60% of MVC, respectively (*p* < 0.05; Figure 3).

**Figure 4:**
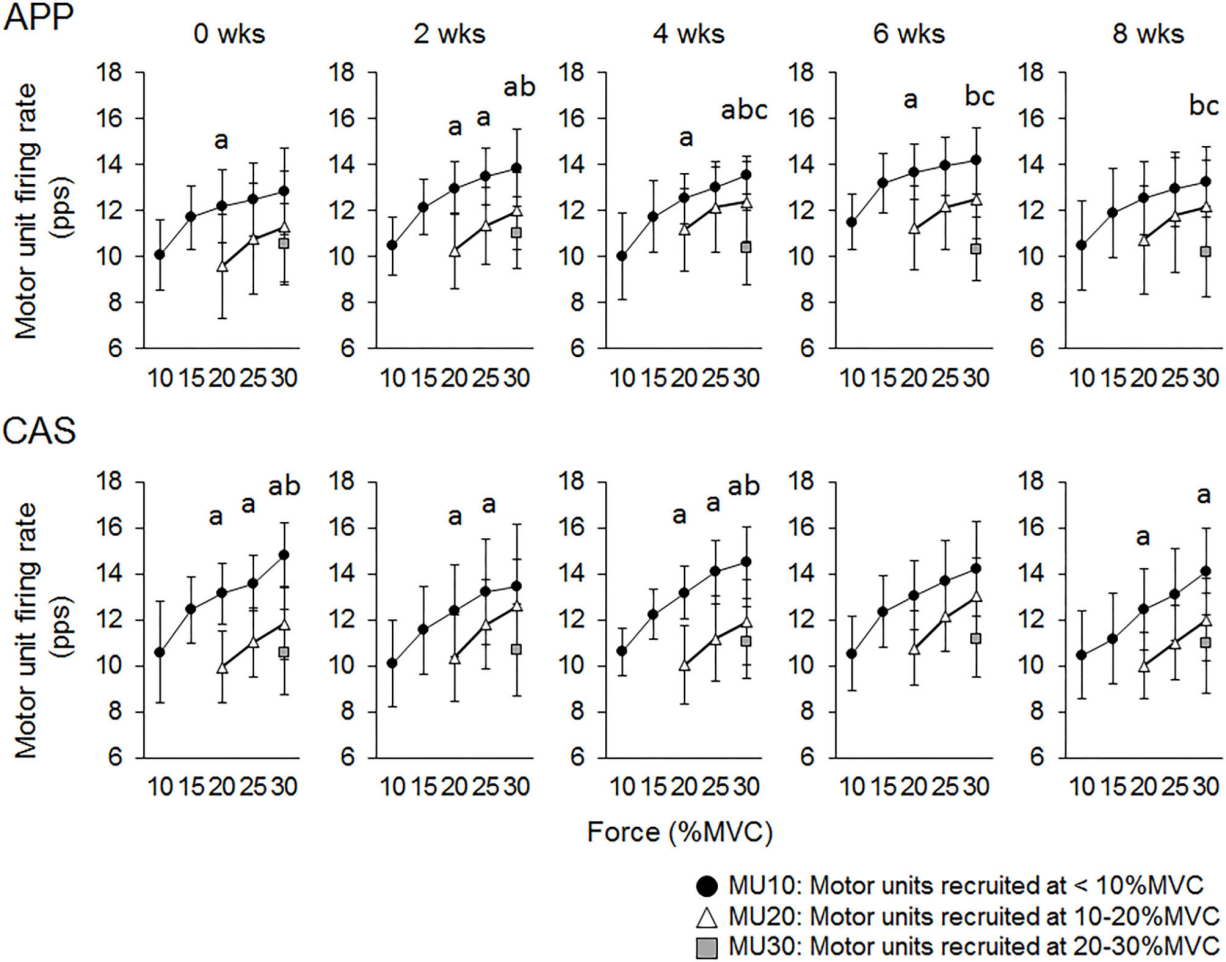
Relationships of firing rates among motor units with different recruitment thresholds during Ramp 30 in groups with ingestions of Alaska pollack protein (APP) and casein (CAS). a, b, and c indicate significant differences for MU10 vs. MU20, MU10 vs. MU30, and MU20 vs. MU30, respectively.

For Ramp 30, at 0 wks significant differences in firing rates were observed between MU10 and MU20 at 20% of MVC (*p* < 0.05) in the APP group and between MU10 and MU20 at 20, 25, and 30% of MVC (*p* < 0.05) and between MU10 and MU30 at 30% of MVC (*p* < 0.025) in the CAS group (Figure 4). Significant differences in firing rates among different motor unit groups that were not observed at 0 wks were noted during the intervention in the APP group: MU10 vs. MU20 at 25% of MVC at 2 wks (*p* < 0.05), MU10 vs. MU30 at 30% of MVC at 2 and 4 wks (*p* < 0.025), MU10 vs. MU30 at 30% of MVC at 2, 4, 6, and 8 wks (*p* < 0.025), and MU20 vs. MU30 at 30% of MVC at 4, 6, and 8 wks (*p* < 0.025) (upper panels in Figure 4), but not in CAS (lower panels in Figure 4).

**Figure 5:**
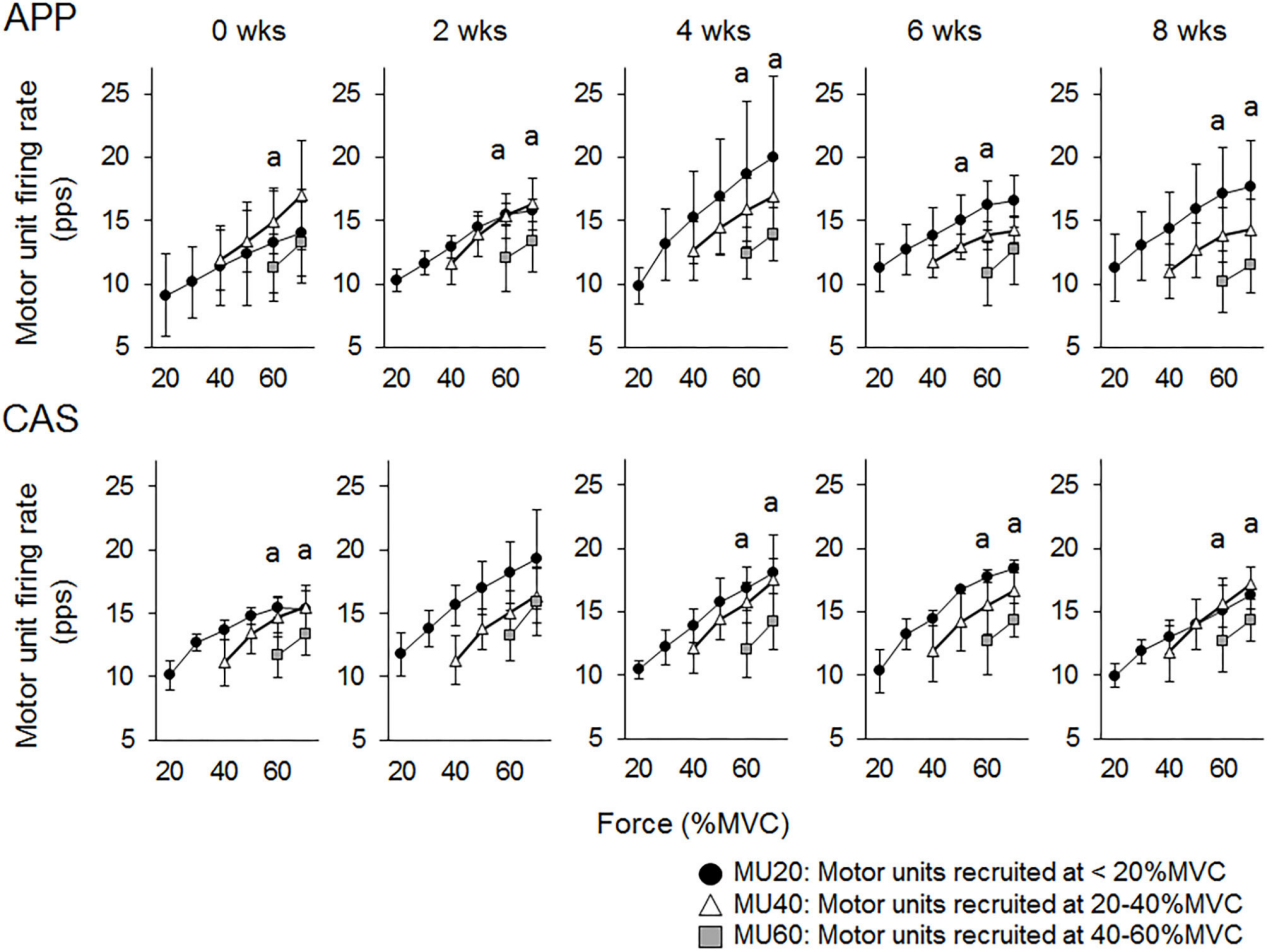
Relationships of firing rates among motor units with different recruitment thresholds during Ramp 70 in groups with ingestions of Alaska pollack protein (APP) and casein (CAS). a, b, and c indicate significant differences for MU20 vs. MU40, MU20 vs. MU60, and MU40 vs. MU60, respectively.

For Ramp 70, significant differences in firing rates were observed between MU20 and MU40 at 60% of MVC (*p* < 0.025) in the APP group and between MU20 and MU40 at 60 and 70% of MVC (*p* < 0.025) in the CAS group (Figure 5) at 0 wks. Significant differences in firing rates among different motor unit groups that were not observed at 0 wks were noted during the intervention in the APP group: MU20 vs. MU40 at 70% of MVC at 2, 4, and 8 wks (*p* < 0.025) and MU20 vs. MU40 at 50% of MVC at 6 wks (*p* < 0.025), in addition to significant differences in firing rates among different motor unit groups that were observed at 0 wks (upper panels of Figure 5). Significant differences in firing rates among different motor unit groups that were observed at 0 wks were also noted at 4, 6, and 8 wks in the CAS group: MU20 vs. MU40 at 60 and 70% of MVC (*p* < 0.025) and no additional pairs of different motor unit groups with significant differences in firing rates during the intervention (lower panels of Figure 5).

## Discussion

The present study investigated the effect of fish protein ingestion on neural and muscular adaptations during resistance training intervention in young adults. Although we did not detect any differences between APP and CAS groups in strength gain and muscular adaptations following 8 weeks of resistance training (Table 2), different neural adaptations such as changes in motor unit firing patterns were noted (Figures 1–4). These results disagreed with those in older adults, in whom APP and CAS ingestions induced muscular and neural adaptations, respectively, with the same level of muscle strength gain during 8 weeks of resistance training (9), supporting our hypothesis.

In the present study, increases in rates of MVC following 8 weeks of resistance training were 38.3% in APP and 34.7% in CAS groups (Table 2), meaning that our training regimen was sufficient to increase muscle strength and induce neuromuscular adaptations. Since we noted no significant increases in muscle volume-related variables, the muscle strength gain in the present study may be primarily due to neural adaptations. In our previous study, increases in muscle volume following resistance training were observed in older adults with APP ingestion, but not in older adults with CAS ingestion (9). These present and previous findings suggest that the same training regimen with APP and CAS ingestion induces strength gains and muscular adaptations that differ in young and older adults. However, we should note that protein intakes in the present study might be insufficient to enhance muscle hypertrophy following resistance training. To maximize muscle protein accretion with resistance exercise, ~1.6 g/kg/day and up to 2.2 g/kg/day of daily protein intakes are recommended (1, 36). Daily protein intakes of the participants from their meals in the present study were 1.3 ± 0.6 g/kg for APP and 1.2 ± 0.6 g/kg for CAS. Taking additional protein intakes by nutritional supplementation which are given in the present study (APP/CAS), total protein intakes are 1.4 ± 0.6 g/kg for APP and 1.3 ± 0.6 g/kg for CAS. Therefore, lack of increase in muscle volume in the present study could be partly explained by inefficient protein intakes.

While no significant changes in the motor unit firing rate were observed in the CAS group, firing rates of some motor units in the APP group were significantly altered following the intervention (Figures 2–5). These changes showed a tendency toward a decrease in the motor unit firing rate from 0 to 8 wks, selectively noted in motor units recruited at over 20% of MVC and at relatively higher force levels (60 and 70% of MVC) (Figure 5). Patten and Kamen (37) also reported decreases in motor unit firing rates during submaximal contraction following resistance training (37), and Sterczala et al. (38) showed decreases in motor unit firing rates in chronically resistance-trained men during submaximal contractions (38). Since we applied the same absolute force in submaximal ramp contractions to record motor unit activation during the intervention, decreases in motor unit firing rates in APP can be interpreted as improvements in neuromuscular efficiency. We also found marked changes in the motor unit firing pattern following the intervention in the APP group (Figure 4). Changes in the motor unit firing pattern were assessed by comparisons of the firing rate among motor unit groups with different recruitment thresholds in the present study, since our previous studies showed that this assessment is useful to estimate motor unit adaptations with aging (21) and training intervention (9, 22). Actually, significant variations in firing rates among motor units with different recruitment thresholds, known as the “onion skin phenomenon” (30, 31), can be found in young adults, but not in older adults (21), and this relationship between firing rates and recruitment thresholds in older adults was changed to the pattern observed in young adults by resistance training (9, 22). Significant differences in firing rates among the motor units with different recruitment thresholds were observed in both APP and CAS groups at 0 wks in the present study (Figures 4, 5). During the intervention, significant differences in firing rates among motor units with different recruitment thresholds that were not observed at 0 wks were newly found at 2, 4, 6, and 8 wks in the APP (upper panels in Figures 4, 5) but not CAS (lower panels in Figures 4, 5) group. This means that characteristic features of motor unit firing patterns in young adults were altered following the intervention when APP was ingested. Based on the changes in relationships of firing rates in different motor unit groups (Figures 4, 5), alterations in the motor unit firing pattern on APP ingestion are due to increases in firing rates of motor units with lower recruitment thresholds and/or decreases in firing rates of motor units with higher recruitment thresholds, and their combinations. Although significant changes were not detected, firing rates of motor units with lower (<20% of MVC) and higher (>20% of MVC) recruitment thresholds tended to increase and decrease during the intervention, respectively, in the APP group (upper panels in Figures 4, 5). On the other hand, a tendency toward decreases in firing rates of motor units with higher recruitment thresholds (>20% of MVC) during the intervention was also noted in the CAS group (lower panels in Figures 4, 5). Therefore, characteristic changes in motor unit firing patterns in the APP group may be partly explainable by the tendency toward increases in firing rates of motor units with lower recruitment thresholds.

However, it is unlikely that protein supplementation such as with APP would act directly on the central nervous system. In addition to modulations of the neural drive via the central nervous system (4), increases and/or decreases in motor unit firing rates following resistance exercise intervention could be explained by changes in muscle contractile properties. Relationships between the stimulation frequency and electrically elicited force are markedly influenced by contractile properties (39), and correlations between motor unit firing patterns and muscle contractile properties (7, 8) have been reported in human skeletal muscles. In previous studies using rat muscle, significant increases in the gastrocnemius muscle weight and fiber diameters were observed by ingestions of APP, but not CAS, during 6- or 8-week interventions (10–12). In a human study, we also showed increases in the muscle volume of older adults with APP ingestion, but not of older adults with CAS ingestion, following 8 weeks of resistance training (9). Muscle proteins are constantly being broken down and synthesized, and these processes are predominantly controlled by physical activity and food consumption (40, 41). We thus considered that processes of muscle protein synthesis could be modified by APP ingestion at the muscle cell level in the present study, while we could not detect significant changes at the whole-muscle level (Table 2). Regarding measurements of muscle contractile properties based on the myosin heavy-chain (MHC) composition, increases in MHC IIA and reductions in MHC IIX following resistance training have been reported in humans (42). Since this change would induce the slowing of contractile properties of fast-twitch fibers, it may explain decreases in firing rates in motor units with higher-recruitment thresholds that exist in both APP and CAS groups (Figures 4, 5). On the other hand, some physiological changes in muscle fibers following resistance training noted in previous studies may explain increases in firing rates of motor units with lower recruitment thresholds observed in the APP group (Figures 4, 5). Łochyński et al. (6) showed a rightward shift in the relationship between the force-frequency curve during electrical muscle stimulation in both fast and slow motor units after volitional weight-lifting in rats (6). This result supports a study by Alway et al. (43) that showed decreases in muscle contraction time in human gastrocnemius muscle following 16 weeks of isometric resistance training (43). However, another study reported no significant changes in contractile properties in any types of muscle fibers following 12 weeks of resistance training in humans (44). In the present study, although physiological processes cannot be determined, the pathway leading to changes in the motor unit firing pattern following resistance training in the APP group could be alterations in muscle contractile properties caused by a modification of muscle protein synthesis processes due to APP intake (10–12). Further studies are needed to clarify the effect of APP ingestion on muscle contractile properties and its relationship with motor unit firing patterns.

Although we investigated the effect of APP ingestion on muscle strength, muscle volume, and motor unit activations in the present and previous study (9, 22), detailed physiological mechanisms/pathways for the effect of fish protein ingestion on neuromuscular systems have not been clarified. Recently, the effects of fish-derived omega-3 fatty acids on muscle strength and volume has seen considerable interest (45, 46). However, the nutritional supplementation for APP which were used in the present study did not include any oils from fish, since fat/oil were removed during the process of making the experimental foods. Therefore, it is difficult to explain the results of the present study by fish-derived omega-3 fatty acids. Also, it should be noted that this study was performed with small sample size and we did not perform sample size calculation prior to the experiment. Moreover, detection of different changes in neural adaptation, such as motor unit firing rate, between APP and CAS may include type I error due to the number of comparisons. Further studies should evaluate the effect of APP on neural and muscular adaptations following resistance training with larger sample number under more statistically controlled conditions.

## Conclusion

We investigated the effect of fish protein ingestion on neural and muscular adaptations during 8 weeks of resistance training intervention in young adults. There were no significant differences in strength gain or muscular adaptations between the groups with APP and CAS ingestions. Significant changes in the firing rate for the motor units with higher recruitment thresholds were observed only in the APP group. Relationships of firing rates among the motor units with different recruitment thresholds were more markedly changed from 2 weeks of intervention in the APP than CAS group, meaning greater changes in motor unit firing patterns on ingesting APP. These results suggest that dietary fish protein ingestion modifies neural adaptation with similar changes in muscle strength and muscular factors in young adults.

## Data Availability Statement

The raw data supporting the conclusions of this article will be made available by the authors, without undue reservation.

## Ethics Statement

The studies involving human participants were reviewed and approved by the Research Ethics Committee of Chukyo University (2015-002, 2016-057). The patients/participants provided their written informed consent to participate in this study.

## Author Contributions

KW and KU made research design. KW and YM collected the data. KW, AH, and YM analyzed the data and reviewed and commented to the first version of the manuscript. KW wrote first version of manuscript. KW, AH, KU, and YM approved the final version of the manuscript. All authors contributed to the article and approved the submitted version.

## Conflict of Interest

KU was employed by Nihon Suisan Kaisha, Ltd. The remaining authors declare that the research was conducted in the absence of any commercial or financial relationships that could be construed as a potential conflict of interest. The authors declare that this study received funding from Nihon Suisan Kaisha, Ltd. The funder had the following involvement with the study: research design.
